# The Spectrum of Manifestations of Severe Acute Respiratory Syndrome-Coronavirus 2 (SARS-CoV2) Infection in Children: What We Can Learn From Multisystem Inflammatory Syndrome in Children (MIS-C)

**DOI:** 10.3389/fmed.2021.747190

**Published:** 2021-10-28

**Authors:** Salvatore Panaro, Marco Cattalini

**Affiliations:** ^1^Rheumatology and Clinical Immunology Unit, University of Brescia and Azienda Socio Sanitaria Territoriale (ASST) Spedali Civili di Brescia, Brescia, Italy; ^2^Pediatric Clinic, University of Brescia and Azienda Socio Sanitaria Territoriale (ASST) Spedali Civili di Brescia, Brescia, Italy

**Keywords:** SARSCoV-2, MIS-C, children, COVID-19, myocarditis

## Abstract

Multisystem Inflammatory Syndrome in Children (MIS-C) is defined as a clinically serious condition requiring hospitalization with fever, multi-system organ disfunction, inflammatory biomarkers increase. The syndrome develops in the context of a probable or ascertained Severe Acute Respiratory Syndrome Coronavirus-2 (SARS-CoV2) infection, but other possible etiologies should be ruled out for definitive diagnosis. On the clinical side, along with the multi-system involvement, myocarditis with heart failure and shock is the most striking feature. Capillary leak is another fundamental feature of MIS-C. In fact, shock and hemodynamic compromise in MIS-C can occur also in the absence of laboratory evidence of myocardial inflammation, with preserved cardiac function and rapid reversibility. Since the first observations of MIS-C patients, it was evident that there is a delay between the peak of adult cases of Coronavirus disease 19 (COVID-19) and the MIS-C peak. Moreover, SARS-Cov2 isolation in children with MIS-C is not always possible, due to low viral load, while positive serology is far more commonly observed. These observations lead to the interpretation of MIS-C as a post-infectious disease. Although the exact pathogenesis of MIS-C is far from being elucidated, it is clear that it is a hyperinflammatory disease with a different inflammatory response as compared to what is seen in acute SARS-CoV-2 infection and that the disease shares some, but not all, immunological features with Macrophage Activation Syndrome (MAS), Kawasaki Disease (KD), Hemophagocytic Lymphohistiocytosis (HLH), and Toxic Shock Syndrome (TSS). Different mechanisms have been hypothesized as being responsible, from molecular mimicry to antibody dependent enhancement (ADE). Some evidence has also been collected on the immunological profile of patients with MIS-C and their difference from COVID-19. This review is focused on critical aspects of MIS-C clinical presentation and pathogenesis, and different immunological profiles. We propose a model where this hyperinflammatory disease represents one manifestation of the SARS-CoV2 spectrum in children, going from asymptomatic carriers to the post-infectious MIS-C, through symptomatic children, a low number of which may suffer from a severe infection with hyperinflammation (pediatric Hyper-COVID).

## Introduction

Coronavirus disease 2019 (COVID-19) is an outbreaking pandemic, threatening public health from at least September 2019. Until now we count at least 127 Million cases through the Globe, with 2,79 Million deaths, as stated by World Health Organization (WHO) (1). Children are less likely to be infected by SARSCoV2 and, even if so, usually develop a mild disease characterized by low-grade fever, abdominal pain and diarrhea and mild upper respiratory tract involvement (2–5). Soon after the first peak of SARSCoV2 in Italy, Verdoni et al. reported on an unusual peak of children presenting with some manifestations of Kawasaki Disease (KD), but with atypical features, as older age at onset, high incidence of cardiogenic shock and myocarditis and abdominal symptoms. In the weeks after, as the SARSCoV2 spread across Europe first and U.S. thereafter more reports came of this hyperinflammatory syndrome possibly related to SARSCoV2 (6–17). This syndrome is nowadays called Multisystem Inflammatory Syndrome in Children (MIS-C) or Pediatric Inflammatory Multisystem Syndrome temporally associated with SARSCoV2 (PIMS-TS) and different case definition criteria have been proposed (11, 18, 19). MIS-C is a serious condition with systemic inflammation, always requiring hospitalization and whose main features are fever, multiorgan dysfunction, elevated acute phase reactants. The syndrome develops in the context of a probable or ascertained SARS-CoV2 infection, but other possible etiologies should be ruled out for definitive diagnosis, as the disease mimics KD shock syndrome (KSS), but also sepsis and Toxic Shock Syndrome (TSS) (20). The epidemiology of MIS-C is still unclear, although it appears to be a relatively rare condition, with an incidence of < 1% in SARS-CoV2-infected children (9). As the number of cases reported is rising, it is not clear which exact mechanism links SARSCoV2 infection to MIS-C, and whether there is clinical overlap between acute severe COVID-19 (Hyper-COVID), MIS-C, and K D. In the lack of controlled trials, the treatment I usually based on the combination of immunoglobulins i.v. (IVIG), systemic steroids and, in the more severe cases anti-cytokine treatments. A literature search through Medline/Pubmed was carried out with different key-words: “SARSCoV2,” “COVID-19,” “MIS-C,” “PIMS-TS,” “Kawasaki Disease SARSCoV2,” “Kawasaki coronavirus,” “Kawasaki like disease,” “SARSCoV2 shock,” “Severe SARSCoV2,” “Severe COVID-19” with and without the filter “children.” We included original studies, reviews, case reports if written in English.

## Pathogenesis

Although a definite model for MIS-C is far from being elucidated, some preliminary evidence is now available.

### The Superantigen Theory vs. Antibody-Dependent Enhancement (ADE)

Epidemiological data showing a peak of MIS-C cases soon after the peak of SARSCoV2 infection in the general population, and the observation that the majority of patients with MIS-C have negative nasopharyngeal swabs but positive serology for SARS-CoV2 point to a post-infective event, whose pathogenesis is still far from clear (10, 21, 22). The disease seems to arise from a dysregulated immune response, leading to a hyperinflammatory state, and endothelial dysfunction, which ultimately causes a capillary leak and multiorgan failure. Notably, there is a clinical and laboratory overlap with some other hyperinflammatory conditions, such as KD (KD), Kawasaki Shock Syndrome (KSS), Haemophagocytic Lymphohistiocytosis (HLH), Macrophage Activation Syndrome (MAS), and Toxic Shock Syndrome (TSS) (23). One hypothesis to explain how SARS-CoV2 may elicit this systemic response is through molecular mimicry, but also a superantigen may play an important role in the process, triggering self-reactive T cells (24). There is evidence that cells from unexposed individuals can respond to S-protein epitopes from SARS-CoV2, supporting the hypothesis of cross-viral immunity from other strains of coronavirus. It is also known that SARS-CoV-2 encodes a superantigen motif that is very similar to another superantigen, known to cause TSS which, as already pointed out, shows remarkable clinical overlapping with MIS-C; the presence of a superantigen domain in selected viral strains of SARS-CoV2 could also explain why MIS-C has been seen in Europe and North America, but not in Asian countries (25). On the other hand, Antibody-Dependent Enhancement (ADE) is another mechanism possibly involved in the pathogenesis of both COVID infection and MIS-C, with crucial clinical implications (26). ADE could explain the clinical overlapping between MIS-C and severe Dengue disease and why some patients, developing high titers of virus-specific antibodies, have a worse clinical outcome (26, 27). ADE has been demonstrated for other coronaviruses: elevated levels of SARS-CoV-1 IgG antibodies in critical SARS cases and anti-S IgG neutralizing antibody responses developed more rapidly after the onset of clinical symptoms in fatal forms compared with recovered cases, leading some to attribute the enhanced tissue damage to ADE (28). One mechanism regarding ADE for coronavirus suggests that the Fc Receptor-antibody complex mimics the viral receptor in mediating viral entry, although this effect seems to be highly dependent on antibody dosage (29). Patients with MIS-C carry higher anti-spike antibodies, compared to children infected by SARS-CoV2 but not developing MIS-C (26). It has also been speculated that, in infants, ADE deriving from maternally acquired SARS-CoV-2 antibodies bound to mast cells can be the triggering mechanism of MIS-C (30). Another possibility is that antibodies directed toward one strain might be not neutralizing or sub-neutralizing for viral infections of other strains and lead to ADE (26). Finally, dissemination of the virus has been demonstrated in children who died from severe COVID-19, with and without MIS-C, suggesting that direct virus replication in different organs has a role in the most severe cases (31).

### The Role of Endothelial Damage

From a pathological point of view, endothelial damage is one of the main features of the disease. This damage may lead to the overexpression of some molecules pivotal for inflammation, such as Toll-Like Receptors (TLRs), IL-1, IL-6, TNF-alpha (32). The role of endothelial damage is suggested by the finding of Burr cells and schistocytes in patients with MIS-C and severe COVID-19, and may explain the occurrence of renal failure and thrombotic microangiopathy seen in some patients successfully treated with eculizumab (20, 33, 34).

### The Role of Dendritic Cells

Dendritic cells can likely be one pivotal target of COVID infection. They are largely distributed in the respiratory tract and through the gut. They are classified as plasmocytoid dendritic cells, producing IFN I, crucial in antiviral response, and classical dendritic cells, interacting with T lymphocytes for priming. A new role for CD147, expressed on lymphocytes, macrophages, and dendritic cells have been suggested for COVID infection. Indeed spike protein can interact with CD147 on dendritic cells and allow virus entry (35). Dendritic cell-specific intracellular adhesion molecule-grabbing non-integrin (DC-SIGN) enhances immune response during viral infections (36). Expression of DC-SIGN or liver/lymph node-specific intercellular adhesion molecule-3-grabbing integrin (L-SIGN) alone has no impact on infection by SARS-CoV2, but amplifies infection of already-permissive cells, i.e., ACE2 expressing cells (37). SARS patients carrying the DC-SIGN promoter−336 G variant, which leads to reduced DC-SIGN protein expression, had lower risk of having severe SARS-CoV2 infection (38). Plasmacytoid dendritic cell-derived type I IFNs is crucial for viral clearance in humans (36, 39). Reduction in the percentage of dendritic cells, mainly of the plasmocytic phenotype, in the peripheral blood of severe patients in both acute and convalescent phases of SARS-CoV-2 infection was also observed (average of 13 and 30 days after symptoms onset, respectively), suggesting a possible defect in type I interferon response as a possible factor for severe disease (40). Further, the percentage of conventional dendritic cells have been found decreased in the resolution phase of MIS-C and dendritic cells also had decreased HLA-DR and CD86, which could indicate impaired antigen presentation to CD8^+^ T cells and priming of naive helper T cells (41).

### Autoantibodies

Preliminary evidence suggests that specific autoantibodies may be responsible for the systemic and organ-specific manifestations of MIS-C. For instance, anti-endoglin (a glycoprotein expressed by endothelium) antibodies were found in several patients affected by MIS-C, but their role is still undefined. There is also preliminary evidence that antibodies against common cold-Coronaviruses could give some protection for MIS-C. IgG antibodies to human coronavirus HKU1 and beta-coronavirus 1 were commonly observed in COVID patients, healthy volunteers and Kawasaki-disease children, but lacked in MIS-C patients. The relevance of this should be still determined but it is possible that the presence of IgG antibodies against common coronaviruses modulates the immune response to SARS-CoV-2 infection and plays a role in the pathogenesis of MIS-C (42).

Differences in antibodies production between children with MIS-C and adults with COVID-19 have been postulated by Weisberg et al. In their study they found distinct antibody profiles in MIS-C, COVID-19, and convalescent plasma donors. MIS-C patients display a restricted antibody response, largely limited to anti-Spike antibodies, with the overall lowest neutralizing activity. On the other hand, patients with ARDS caused by primary infection show the highest overall levels and the most prominent neutralizing activity. The MIS-C cohort lacked anti Nucleocapsid (Anti-N) antibodies, letting one think to a weaker immune response in this subset of patients, almost not neutralizing, although these results are not definitive (43).

## Clinical Presentation

### General Features

MIS-C is believed to occur 2–6 weeks after SARS-CoV2 infection, although definite demonstration of a preceding infection in children with MIS-C is not always possible. Data from the first case series showed that the majority of patients with MIS-C are positive only for SARS-CoV2 serology, almost a third of the patients resulted positive on both serology and nasal swabs polymerase chain reaction (PCR), while a minority of patients (around 5%) are negative on both SARS-CoV2 antibodies and PCR (6, 10, 11, 22, 44). This delay between SARS-CoV2 infection and MIS-C onset is testified also by the delay that has been described in different countries between COVID-19 peaks in the general population and MIS-C occurrence and may justify the higher viral cycle thresholds of MIS-C patients compared to severe COVID cases observed (10, 20, 22).

MIS-C usually has an abrupt onset with high spiking fever and signs and symptoms of systemic involvement. The clinical picture may be wide, in terms of organ manifestations and severity. In a recent systematic review, the commoner symptoms were gastrointestinal (71%), with the occurrence of abdominal pain (36%), diarrhea (27%), and vomiting (25%), followed by mucocutaneous manifestations (skin rash, strawberry tongue, dried-cracked lips, conjunctivitis) (33, 34). Patients may present with shock in a high percentage of cases, from 30 to 70%, depending on different case series published. Shock is most probably determined by a concurrence of heart failure and capillary leak syndrome and requires intensive care treatment (7, 16, 45, 46). Heart involvement is, indeed, one of the most striking features of MIS-C as further discussed. Neurological involvement is quite common, described in up to 20% of affected patients, with varying severity, from irritability and meningismus to severe encephalopathy (47). Patients may also present with kidney failure. Most notably, respiratory symptoms are seldomly described, and this may help to differentiate MIS-C from severe COVID-19, as further discussed (48).

### Cardiovascular Involvement

Cardiac involvement is very common in MIS-C, and one of the key features to distinguish this disease from severe COVID-19. Myocarditis seems to be the most common cardiac manifestation: more than a third of reported patients in the literature showed depressed cardiac function with variable severity, and this was the main cause for ICU admission (48). Mild to moderate mitral regurgitation and tricuspid regurgitation, but also some localized contractility defects are the most common findings on echocardiography. In those cases where MRI was performed, it showed myocardial edema with late-gadolinium enhancement (46). Markers of cardiac involvement, namely Troponin-T, brain natriuretic peptide P (BNP) and N-terminal-prohormone BNP (NT-proBNP) were raised in up to 77% of patients (46). Different mechanisms have been proposed to cause heart involvement, from myocardial edema to myocardial stunning, but also myocardial necrosis (49, 50). An intense inflammatory state, but also transitory ischemic states secondary to the hypoperfusion and hypotension (in the most severe MIS-C cases) may be possible determinants of the cardiac stunning. MIS-C with myocarditis shows a stronger inflammatory profile compared to patients without myocarditis. Eleven cytokines and chemokines, including CSF2, CCL2, IL-6, CXCL10, FLT3L, 177 VEGF, TGF-A, IL-1RA, PD-L1, CX3CL1, TGF-B1, were found to be higher in MIS-C with severe myocarditis (51). In contrast, a slightly higher expression of IFN-α2 and IL-17A was found in MIS-C without myocarditis (51). Another possible mechanism of damage is the direct invasion of SARS-CoV2 in the heart: post-mortem biopsy analysis in few children showed myocarditis, endocarditis, pericarditis with necrosis of cardiomyocytes; the presence of viral particles in endothelium, myocardium, myocardial macrophages, together with lung and kidney microthrombi (16, 52).

Other less common findings in children with MIS-C are pericarditis, in up to 20% of patients, and coronary artery aneurisms (CAAs), which are reported in up to 25% of patients (44, 46). The occurrence of CAAs in patients with MIS-C is intriguing, as CAAs are a classic feature of Kawasaki Disease (KD) and, together with some mucocutaneous manifestations that are present in some patients, are the main feature suggesting common pathogenesis, if not a continuum of disease, between MIS-C and KD. Interestingly, the incidence of CAAs was the same among three different categories of patients with MIS-C, where two of the three categories had no other common manifestations of KD (11). The outcome of cardiac manifestations seems to be very good in patients with MIS-C, as the majority of cases show resolution within few weeks, probably for the aggressive treatment the majority of patients receive (53).

### Laboratory Findings

Lab tests in patients with MIS-C testify the systemic inflammation. The most common findings are leukocytosis with neutrophilia, elevated inflammatory markers (CRP, ESR, fibrinogen, procalcitonin), mild anemia, in up to 90% of patients. Some patients may exhibit lymphopenia, although lymphocyte levels are usually higher than in patients with acute COVID-19. Finding high Troponin T and/or B natriuretic peptide (BNP) or N-terminal pro-BNP (NT-pro-BNP) may suggest cardiac involvement (48). Liver enzymes may be found elevated and the most inflamed patients may satisfy Macrophage Activation Syndrome criteria, with thrombocytopenia, markedly elevated ferritin, hypofibrinogenemia, elevated lactate dehydrogenase (6, 22). Hypoalbuminemia and prolonged PT and aPTT may be found. Higher inflammatory markers and markers for cardiac involvement seems to correlate with a poorer prognosis (54).

As already pointed out, evidence of infection from SARS-CoV2 is present in almost 60% of cases, through positive PCR on nasopharyngeal swabs or, more commonly, positive serology. Noteworthy, as per case definitions, in case PCR o serology is negative, patients must have a positive history of SARS-CoV2 exposure.

### Imaging

Hameed et al. described a case series of 35 children with MIS-C. Chest radiography can be negative or can show peri-bronchial cuffing and perihilar interstitial thickening (34%), perihilar airspace opacification (31%). Interestingly, these findings associate with cardiac dysfunction (12). In some cases, a focal perihilar consolidation at admission, as well as small bilateral pleural effusions and atelectatic changes, changing within days from site to site were described (55).

Thoracic computer tomography (CT) imaging was performed when embolism was suspected, due to raised values of D dimer and fibrinogen. Basal consolidation with collapse (39%) and pleural effusions (30%) were the most common findings (55).

As MIS-C has some overlapping features with KD, cardiac CT was performed in case of myocardial dysfunction in 30 of the 35 children (80%) and showed abnormal coronary artery aneurysms in 6 (20%). Aneurysms ranged from very mild single coronary artery dilatation (e.g., left anterior descending artery diameter of 4.3 × 4.1 mm and z score of +2.7) to large aneurysms affecting more than one coronary artery (left anterior descending artery diameter of 6.5 × 7.7 mm and *z* score of +13.9 in one child) (55). Heart magnetic resonance imaging (MRI) showed signs of diffuse myocardial edema and hyperemia with no focal myocardial necrosis or fibrosis (40, 55).

### Outcome

Although MIS-C may have an abrupt onset requiring intensive care management, the global outcome is generally favorable. According to a systematic review, the duration of hospitalization was 4–13 days (median, 7 days), and intensive care was required in 68% of patients. Inotropic support was required in 40%, mechanical ventilation was required in 15%, and ECMO was required in 2.7%. The fatality rate was reported to be 1.7% in the US and 1.4% in Europe (56). Among the studies that reported outcomes at discharge (13) or during follow-up, almost all patients with cardiac involvement experienced nearly full recovery of left ventricular function and normalization of cardiac inflammatory markers except for mild cardiac dysfunction observed in nine patients at discharge in one study (57–60).

When compared with classic KD, MIS-C patients had a worse left ventricular systolic and diastolic function. The strongest predictors associated with myocardial injury in MIS-C patients were globulin longitudinal strain (GLS), global circumferential strain (GCS), left atrial strain (LAS), and longitudinal strain of the right ventricular free wall (RVFWS), with an odds ratio: 1.45 [95% confidence interval (CI): 1.08–1.95], 1.39 [95% CI: 1.04–1.88], 0.84 [95% CI: 0.73–0.96], 1.59 [95% CI: 1.09–2.34], respectively) (61). Higher inflammatory markers and markers for cardiac involvement seems to correlate with a poorer prognosis (54).

## Treatment

To date, the majority of patients with MIS-C have been treated with a combination of systemic corticosteroids and high-dose i.v. immunoglobulins (IVIG). This is most certainly due to the clinical overlap between MIS-C and KD. On this basis, some scientific societies proposed guidance for management and treatment of MIS-C and, although with slight differences among them, they generally suggest tailoring the treatment on the patient clinical picture and general management with the use of IVIG alone in patients with less severe disease, adding systemic corticosteroids (1–2 mg/kg/day i.v.) in patients with evidence of shock (62–64). Pulse methylprednisolone is considered an option for the most severe patients by some societies. Finally, as for KD itself, Anakinra has been proposed for the treatment of refractory cases, or on top of corticosteroids and immunoglobulins at disease onset in the most severe patients (i.e., patients needing ICU admittance, with signs or symptoms of secondary HLH) (65). Few data are available to address the real efficacy of different treatments. In a recent study by Son et al., the initial treatment with IVIG plus glucocorticoids was associated with a lower risk of new or persistent cardiovascular dysfunction than IVIG alone, while McArdle et al. found no evidence that recovery from MIS-C differed after primary treatment with IVIG alone, IVIG plus glucocorticoids, or glucocorticoids alone (53, 66). These discrepancies are most probably due to the retrospective nature of the studies and to patients heterogeneity. Tocilizumab has also been used in different case series but no specific trials are available (44, 49).

Other ancillary treatments regarding thrombotic risk and inotropic support must be evaluated case by case. Acetylsalicylic acid should be given in case of coronary abnormalities, as it is for KD (64). Prophylactic low molecular weight heparin should be considered in children with MIS-C, given the high inflammatory state and stratifying the thrombotic risk based on D-Dimer levels and the presence of other pro-thrombotic risk factors (67). Inotropic support is another important issue because of capillary leak complicating MIS-C (68). Finally, therapy with eculizumab should be considered in case of acute kidney failure and evidence of microangiopathy (34).

## Hot-Topics for Discussion

### MIS-C, KD, or Severe COVID-19? The Spectrum of Clinical Manifestations of SARS-CoV2 Infection in Children

The clinical picture of MIS-C is quite obvious in the majority of cases; still, the disease has many overlaps with other conditions, such as KD, Toxic Shock Syndrome (TSS), and sepsis (see Table 1). Case definition criteria have been proposed for the prompt identification and treatment of suspected cases, but their sensitivity has never been evaluated. There is now evidence that patients with MIS-C may satisfy also Kawasaki Disease criteria and that some patients with acute COVID-19 may have severe disease, with some features of systemic inflammation, and possibly satisfying criteria for MIS-C (here referred to as Hyper-COVID) (22, 69). To close the loop, clear-cut Kawasaki Disease cases, without the classic features of MIS-C (such as shock and myocarditis) and positive nasopharyngeal swabs for SARS-CoV2, have been described (70).

**Table 1:** Main clinical and laboratory differences between MIS-C, Hyper-COVID, KD, TSS, and MAS (macrophage activation syndrome).

	**MIS-C**	**Hyper-COVID**	**KD**	**TSS**	**MAS**
Age	Older children (>8 y/o)	Adolescents	Young Children (<5 y/o)	Older children (>10 yrs)	Variable
Sex	Male	Equal	Male	Equal	Equal
Ethnicity	Non-African black	Equal	Asian	Equal	Equal
Skin involvement	KD-like >50% cases	Uncommon (rash)	Typical	Very common (erythema)	None
G.I. involvement	Severe abdominal pain/diarrhea common	Abdominal pain/diahhrea possible	Uncommon	Common	Uncommon
Respiratory Involvement	Uncommon	Severe low respiratory tract Common	Uncommon	Uncommon	Uncommon
Neurologic involvement	Irritability/ meningismus Very common to common	Uncommon	Irritability very common Meningismus uncommon	Common	Severe Common
Kidney Involvement	Possible AKI	Uncommon	Uncommon	Common	Uncommon
Hepatosplenomegaly	Common	Uncommon	Possible	Uncommon	Very common
Lymphadenopathy	Possible	Uncommon	Very common	Uncommon	Very common
Heart Involvement	Very common Myocarditis/pericarditis/CAAs	Uncommon	Common CAAs	Common (myocarditis)	Uncommon
Shock	Common	Uncommon	Possible	Common	Uncommon
Lymphocyte count	Normal to low	Very low	Normal	Normal to high	Normal to low
Neutrophil count	Normal to high	Normal	Normal to high	high	Normal to low
Platelets	Normal to low	Normal to low	Normal	Normal	Normal to low
LFTs	Normal to high	Normal to high	Normal to High	Normal	High to very high
Triglycerides	Normal	Normal to high	Normal	Normal	High
Fibrinogen	Normal to low	Normal to low	Normal to high	Normal to high	Very low
Ferritin	Normal to high	Normal to high	Normal to high	Normal to high	Very high
CRP	High to very high	High to very high	High	high	High to very high

Although pediatric COVID-19 is generally a benign disease a minority of patients require ICU admittance for severe manifestations (15, 71). Bhumbra et al. reported on few patients with severe COVID-19, requiring ICU admission, and compared their characteristics with less severe patients. Patients requiring ICU were older, with longer disease duration before admittance, worse respiratory parameters and evidence of intense systemic inflammation with lower WBC, platelets, and higher inflammatory markers (69). When reviewing MIS-C cases in the US during the march to July 2020 period, Godfred-Cato et al. were able to distinguish three different categories of patients: a third of the patients had a higher incidence of multiorgan involvement, with the cardiovascular and intestinal systems being almost constantly affected. Those patients had also the highest incidence of shock, higher inflammatory markers, and more commonly showed increased Troponin, BNP, or pro-BNP. SARS-CoV2 serology was positive in almost all the children in this category. The second category of patients, encompassing almost a third of the studied population, included younger children, with a higher incidence of mucocutaneous manifestations and lower incidence of multisystem involvement. Children from this category had also a lower incidence of shock, myocarditis, and most commonly satisfied Kawasaki criteria. SARS-CoV2 serology was positive in 2/3 of the patients, with a third having also PCR positivity. Finally, the remaining third of the patients had a higher incidence of respiratory symptoms and severe respiratory involvement, with higher fatality rates and SARS-CoV2 swabs were positive in a significantly higher percentage of children. The authors themselves hypothesize that this third category of patients most probably comprises children with acute severe COVID-19, satisfying also MIS-C criteria (11). Feldstein et al. recently published their research where they recruited more than 1,100 children with SARS-CoV2-related diseases, comparing those with MIS-C (as per CDC criteria) from those with severe acute COVID-19 (as per a pre-defined set of criteria). Patients with MIS-C were significantly younger, with a higher incidence of non-Hispanic Black ethnicity, lower incidence of comorbidities, higher incidence of cardiovascular involvement without respiratory involvement, and higher incidence of mucocutaneous manifestations. Patients with MIS-C also had higher inflammatory markers than patients with acute COVID-19 (44).

Taken together all this evidence seems to suggest that SARS-CoV2 may determine in children a spectrum of diseases: on one end of the spectrum, there are the majority of children, that remain asymptomatic or, develop a very mild disease, which is clinically characterized by a low-grade fever and mild gastrointestinal and respiratory involvement. A lower number of ACE2 receptors in the high respiratory tract of children, but also the so-called “trained immunity” theory have been recalled as responsible for this benign course of SARS-CoV2 infection (72–74). Moving through the spectrum of diseases there is then the minority of children that develop Hyper-COVID. These are usually older children, usually with comorbidities. Severe respiratory involvement, often requiring ICU treatments and high fatality rates are the main characteristics of these children, which usually have positive nasopharyngeal swabs for SARS-CoV2. At the other end of the spectrum, there is the post-infection disease, called MIS-C. High incidence of shock, cardiac and gastrointestinal involvement, very intense systemic inflammation, and racial predisposition are key features of this form. Patients with MIS-C may have some features of KD, mainly the mucocutaneous manifestations and CAAs formation so that some of them, usually the younger ones, satisfy KD criteria. Finally, there is also the possibility to have *bona-fide* KD triggered by SARS-CoV2 (70). The high incidence of CAAs in the cohort of patients studied, underlie that the two diseases share some common pathogenetic mechanisms.

Many attempts are directed toward the identification of different immunological profiles to distinguish MIS-C and severe COVID (Table 2). According to a recent study, in MIS-C marked thrombocytopenia, neutrophilia, a higher neutrophils/lymphocytes ratio and higher levels of Myeloperoxidase (MPO) were found compared to COVID-19 (75). C-Reactive Protein plasma levels were found to be higher in MIS-C rather than in COVID-19 children (75). In MIS-C an important reduction of plasmocytoid dendritic cells was found, with a proinflammatory cytokine profile characterized by high levels of IL-6, CXCL8, CCL2, CXCL9, and CXCL10 (75). Acute COVID disease cytokine profile is characterized by high IFNα levels with respect to MIS-C, while IFN-γ was undetectable in both processes. This data is congruent with a reduction in plasmocytoid dendritic cells encountered in MIS-C, as they produce large amounts of IFNα; nevertheless, others found an increase in IFN-γ (20, 75). The role of many molecules, including IFN-γ, was studied by Smith et al. (76). They found an important role for CXCL9, a monokine induced by IFN-γ. Increased levels of CXCL9 were correlated with the severity of MIS-C (76). Indeed, CXCL9 could possibly allow distinguishing MIS-C from KD. The optimal CXCL9 value to distinguish MIS-C from KD was determined to be 535 pg/mL with a sensitivity of 93% and specificity of 100% (76). Furthermore, CXCL9 followed the clinical picture, as it decreased after administration of immunomodulators (76). Other authors pointed out that the sum of IL-10 and TNF-α levels allowed to distinguish MISC from severe COVID-19 presentations, but not between severe and mild MIS-C (20). SARS–CoV2 reverse transcription polymerase chain reaction (RT-PCR) cycle thresholds were found to be low in COVID patients and high in MIS-C patients (20). Soluble C5b-9 (sC5b-9), instead, has been suggested to be useful to distinguish severe COVID-19 from mild COVID-19, but not severe COVID-19 from MIS-C (20).

**Table 2:** Merging knowledge from different studies.

**MIS-C**	**COVID**
^*^MIS-C with severe myocarditis	
High levels of CSF2, CCL2, IL-6, CXCL10, FLT3L, 177 VEGF, TGF-A, IL-1RA, PD-L1, CX3CL1, TGF-B1 (51)	High IFNα (75)
Neutrophilia (75)	
Decreased NF-KB inhibitors^*^ (51)	
Thrombocytopenia (75)	
Higher ratio neutrophils/lymphocytes (75)	
Increased S-100 proteins^*^ (51)	
High MPO expression (80)	
Increased NF-KB expression^*^ (51)	
Low plasmocytoid dendritic cells (75)	
Decreased MHC II expression^*^ (51)	
High levels of IFNγ, IFNα2, IL-17A, TNF-α, IL-10, Granzyme B (51)	Lower levels of IFNγ, IFNα2, IL-17A, TNF-α, IL-10, Granzyme B compared to MIS-C (51)
High levels of IL-10 + TNF-α (20)	
High levels of Hypoxia induced response (HIF1-α)^*^ (51)	
High levels of IFNα2 and IL-17A^*^ (51)	
High SARS–CoV2 RT-PCR cycle threshold (20)	Low SARS–CoV2 RT-PCR cycle threshold (20)
Restricted antibody response, mainly anti-Spike antibodies, lower neutralizing activity (43)	Higher levels of specific antibodies with the most prominent neutralizing activity (43)
Lack of anti-N antibodies (43)	Presence of anti- N antibodies (43)
Overexpression of interferon induced genes (51)	

* *means MIS-C with severe myocarditis*.

### Post-vaccine Myocarditis: Is There a Link With MIS-C?

Some reports have been published since May 2021 on the occurrence of myocarditis and pericarditis in adult patients receiving the mRNA vaccines. Myocarditis seems to occur mainly in people under 30 years of age and is usually very mild (77). More recently, Marshall et al. reported on seven adolescents (from 14 to 19 years old) who developed myocarditis soon after SARS-CoV2 vaccine (78). Although there was no evidence of a causal relationship between SARS-CoV2 vaccination and the occurrence of myocarditis, the observation that myocarditis is one of the main features of MIS-C whose pathogenesis may be linked to the production of autoantibodies, there have been concerns that SARS-CoV2 vaccines may be related to MIS-C. This is in contrast with the funding that all seven adolescents lately reported had no evidence of acute SARS-CoV-2 infection and did not fulfill criteria for MIS-C, also, myocarditis was generally mild and all patients recovered without sequelae. Myocarditis has been linked to other vaccines, smallpox in particular, but a possible link between SARS-CoV2 vaccine and MIS-C could not be excluded by now and only further understanding of MIS-C pathogenesis would lead to final conclusions (79). It is crucial to underline that, at the time Marshall et al. reported on the 7 adolescents with myocarditis, more than 2.5 million doses of the Pfizer/BioNTech vaccine had been delivered to adolescents 12–15 years old and 4 million doses were given to 16–18 years since FDA EUA approval. As 4 million COVID-19 cases have been diagnosed in children under 18 in the US that resulted in over 15,000 hospitalizations and between 300 and 600 deaths, it is clear that, by now, the benefits of vaccination far exceed the risks of rare adverse events (80).

## Conclusions

MIS-C is a post-infectious severe disease occurring in children with a SARSCoV2 previous contact. As the definition and clinical characteristics may overlap with severe acute SARSCoV2 infection (referred here as HyperCOVID), but also with other hyperinflammatory conditions (such as KSS, sHLH, TSS) the careful evaluation of both clinical features and laboratory markers are needed before a final diagnosis is established. To date, the best treatment strategy seems to rely on the variable association of systemic corticosteroids, IVIG and anti-IL-1 treatments, tailored on an individual basis depending on the disease severity. Future research should be focused on a better definition of the therapeutic strategy, possibly with randomized trials. A very crucial point to further explore is the pathogenesis of the disease, and in particular of the possible role of anti-SARSCoV2 antibodies, also to rule/out the possibility of vaccine-induced MIS-C or MIS-C like manifestations.

## Author Contributions

All authors listed have made a substantial, direct and intellectual contribution to the work, and approved it for publication.

## Conflict of Interest

The authors declare that the research was conducted in the absence of any commercial or financial relationships that could be construed as a potential conflict of interest.

## Publisher's Note

All claims expressed in this article are solely those of the authors and do not necessarily represent those of their affiliated organizations, or those of the publisher, the editors and the reviewers. Any product that may be evaluated in this article, or claim that may be made by its manufacturer, is not guaranteed or endorsed by the publisher.
